# Genomic diversity of *Trypanosoma cruzi* clinical isolates: a retrospective analysis

**DOI:** 10.1016/j.lanmic.2026.101446

**Published:** 2026-08-25

**Authors:** Jill M C Hakim, Sneider Alexander Guiterrez Guarnizo, Angel Duran, Edith Silvia Malaga Machaca, Carolina Duque, Lulu Singer, Rony Colanzi, Jacqueline E Sherbuk, Caryn Bern, Robert H Gilman, Louisa A Messenger, Monica R Mugnier

**Affiliations:** Department of Molecular Microbiology and Immunology, Johns Hopkins Bloomberg School of Public Health, Baltimore, MD, USA (J M C Hakim MPH PhD, A Duran BS, L Singer ScM, M R Mugnier BS PhD); Department of International Health, Johns Hopkins Bloomberg School of Public Health Baltimore, MD, USA (S A G Guarnizo PhD, Prof R H Gilman MD); Infectious Diseases Research Laboratory, Department of Cellular and Molecular Sciences, Universidad Peruana Cayetano Heredia, Lima, Peru (E S M Machaca MS); Department of Pathology, Johns Hopkins School of Medicine, Baltimore, MD, USA (C Duque PhD); Department of Pathology, Hospital Japonés Santa Cruz, Bolivia (R Colanzi MD); University of South Florida Morsani College of Medicine, Division of Infectious Diseases and International Health, Tampa, FL, USA (J E Sherbuk MD); Department of Epidemiology and Biostatistics of the University of California, San Francisco School of Medicine, San Francisco, CA, USA (Prof C Bern MD); Department of Environmental and Global Health, Parasitology and Vector Biology Laboratory, University of Nevada, Las Vegas, NV, USA (L A Messenger PhD)

## Abstract

**Background:**

*Trypanosoma cruzi* causes Chagas disease, a poorly understood and clinically heterogeneous disease. Recent work has shown that parasites adapted to laboratory conditions exhibit genomic variation; however, little is known of the extent of genomic diversity among clinically isolated specimens. In this study, we aimed to describe genomic diversity within a set of clinically isolated strains.

**Methods:**

We isolated 15 *T cruzi* specimens from three cross-sectional clinical studies of Chagas disease in Bolivia. We sequenced the genome of each strain, estimated parasite genetic lineage, genomic population structure, and regions of copy number plasticity, and identified gene conversion events. We generated and annotated genome assemblies of each isolate and compared the gene repertoires encoding the multigene families (MGFs) of *T cruzi*.

**Findings:**

We identified parasites from two genetic lineages in this collection of clinical isolates. Our analysis revealed evidence of genomic instability, copy number variation, and regions with loss of heterozygosity. We also discovered a set of MGF members present in all the clinically isolated parasite genomes and absent from all the laboratory-adapted strains assessed in our comparative dataset, regardless of parasite lineage. MGF repertoires from genomes of the same lineage were more conserved in content among field-isolated specimens than among culture-adapted strains.

**Interpretation:**

This study provides, to the best of our knowledge, the first whole-genome sequencing data for TcV parasites isolated from human participants with Chagas disease. Our analysis of these genomes revealed genomic instability; moreover, we observed a set of genes that are present exclusively within clinical isolates and absent in laboratory-adapted strains, indicating evolutionary pressure for the maintenance of these genes in natural hosts. These findings highlight the limitations of genetic studies focused exclusively on laboratory-adapted parasite strains, which could miss genomic features of *T cruzi* that might be important for clinical infection.

## Introduction

*Trypanosoma cruzi*, the causative agent of Chagas disease, is estimated to infect 6·3 million people and kill 7000 people each year.^1^ *T cruzi* is transmitted primarily via the triatomine vector, although congenital transmission, oral infection, and blood transfusions are also appreciable sources of new cases. Chagas disease is a phenotypically heterogeneous disease; 30% of infected individuals will develop potentially fatal cardiomyopathy, others might develop megacolon or megaoesophagus, and most will remain infected without clinical symptoms.^2^

This phenotypic diversity is accompanied by substantial genomic variability. Parasites are grouped into discrete lineages based on their nuclear genome sequences, referred to as discrete typing units (DTUs; TcI–TcVI), which are grouped into clades that define the genomic ancestry of each strain.^3^ TcI and TcII are the most genetically distant from each other, whereas TcV and TcVI are both hybrids of TcII and TcIII. Substantial work has been performed on parasite diversity via simple genetic typing;^4^ however, relying solely on DTU classification to describe *T cruzi* genomic variability potentially obscures important genomic variation that might contribute to clinical presentation. Although TcV strains are especially common in areas with high clinical burden of Chagas disease, such as Bolivia, and are implicated in congenital transmission, substantial evidence of biologically relevant variability is seen within this DTU.^4^ Despite this evidence, little work has been performed to characterise the range of genomic diversity within TcV strains.

The extensive genomic variability even within parasite lineages is at least partially the result of the genomic plasticity of *T cruzi*. Copy number variability has been observed in field isolates from vectors, and serial passage in culture has experimentally shown the parasites’ capacity for copy number plasticity.^5,6^ *T cruzi* genomes contain core regions that are largely syntenic across trypanosomatids; however, these regions are interrupted by extensive genomic regions encoding large multigene families (MGFs).^7^ Many of these MGFs, including mucins, mucin-associated proteins, trans-sialidases, discrete gene family-1, glycoprotein-63, and retrotransposon hotspot proteins, are highly immunogenic and largely surface exposed and are thus thought to contribute to pathogenesis.^8-13^

Although *T cruzi* genomes are highly variable and diverse even within individual parasite lineages, these complexities have only been studied in strains adapted to laboratory culture. This focus is largely owing to the challenges associated with generating sufficient genetic material required for whole-genome sequencing of clinically isolated strains. Parasites are typically undetectable in blood during the most common chronic stages of Chagas disease. Even when parasites are detectable, isolating them from clinical specimens is a challenging and time-intensive process. The few previous studies of clinically isolated specimens have shown that clinical isolates are phenotypically distinct in terms of growth rate and drug response from laboratory-adapted strains.^14,15^ Nevertheless, the possible genomic basis for these phenotypic differences remains unclear.

In this study, we aimed to investigate the relationship between genetic variability in *T cruzi* and its phenotypic heterogeneity. We isolated and sequenced *T cruzi* parasites from clinical cases of Chagas disease across three independent clinical studies, representing different infection contexts, to identify genetic features important for human infection.

## Methods

### Study design

Blood samples were collected from three ongoing clinical studies based in Santa Cruz, Bolivia, as part of a retrospective genomic study. The studies were approved by Johns Hopkins School of Public Health under institutional review board numbers IRB00004303 for opportunistic infections during HIV infection, IRB00005598 for cardiomyopathy, and IRB00002644 for congenital studies and by local review boards at Hospital Universitario Japonés, Asociación Benéfica Proyectos en Informatica (PRISMA), and Universidad Catolica Boliviana (Santa Cruz, Bolivia). A description of a larger congenital cohort has been reported by Kaplinski and colleagues and by Messenger and Bern, and the cardiomyopathy study has been described by Okamoto and colleagues and Sherbuk and colleagues.^16-19^ The congenital and cardiomyopathy studies were designed as longitudinal, although only cross-sectional samples were successfully isolated and included in this study. The opportunistic infection study design was cross-sectional. The STROBE checklist is included in appendix 1 (pp 23-26).

### Participants

Samples that were included in this study were isolated from participants of the aforementioned ongoing epidemiological studies. All participants provided written informed consent. From these studies, haemoculture was attempted in a convenience sample of 147 individuals with confirmed positive *T cruzi* serology. A detailed description of the haemoculture is provided in appendix 1 (p 17). All culture attempts were noted, and there were no missing isolate data.

### Procedures

15 mL of venous blood was collected in EDTA-coated tubes from patients who were *T cruzi* PCR cruzi1 positive or cruzi2 positive.^20^ Samples were centrifuged at 1200 *×g* for 10 min at 4°C. All but 0·5 mL of plasma and packed red cells were aspirated from the supernatant, and 8 mL of supplemented RPMI-1640 was subsequently added to the packed red cells. Samples were centrifuged again at 1200 *×g* for 10 min; the supernatant was discarded, and 2 mL of packed red cells was distributed across three tubes (to avoid risk of contamination) filled with biphasic media containing blood agar (1·4% blood agar [Sigma 70 133], 0·5% trypticase peptone [Sigma T9410], 0·6% purified agar, and 0·6% NaCl) and overlay (0·9% NaCl and 150 μg/mL gentamicin [Sigma G1522]). Biphasic media cultures were incubated at 24–28°C, and cultures were examined for flagellates at 30, 60, 90, 120, 150, and 180 days. Once parasite growth was observed, cultures were transferred to liver infusion tryptose medium. After approximately 10 days of cultivation, and once 10^3^ parasites were observed, three washes with phosphate-buffered saline were performed by centrifugation at 2000 *×g* for 15 min. The resulting pellet was resuspended in 10% dimethyl sulfoxide in inactivated fetal bovine serum and stored at −80° C until further processing.

### Extraction and sequencing

Samples were expanded and cryopreserved in Bolivia as previously described and stored at −80°C for, in some cases, over a decade.^21^ After receiving cryopreserved pellets in the USA, all parasite cultures were non-viable upon thawing and thus could not be expanded further for cloning or the isolation of sufficient DNA for long-read sequencing. Given the low input material quantity, Illumina short-read sequencing, which requires only nanograms of input DNA, was used. DNA was extracted using the QIAamp DNA blood kit for genomic DNA extraction following the manufacturer’s instructions (Qiagen, 51 104, Hilden, Germany), and Illumina libraries were prepared using the NEB Nextera XT DNA Library Preparation Kit (NEB FC-131-1096, Ipswich, MA, USA). Libraries were sequenced on an Illumina NovaSeq X at the Johns Hopkins Genetic Resources Core Facility. Sequencing adaptors were trimmed using default parameters with BBduk (v39).^22^

### Statistical and bioinformatic analyses

All details of short-read whole-genome assembly, reference-based analysis, and methods used in the manuscript are presented in appendix 1 (pp 14-17).

The primary outcome of genomic diversity between the genomes of clinically isolated parasites, including copy number variation heterozygosity and genetic population structure using an single nucleotide polymorphism matrix, was assessed.

Statistical analysis was performed in R v.4.2.2. All code to run analysis and generate figures are available at GitHub. Raw sequencing data are available in the Sequence Read Archive under BioProject PRJNA1271783.

### Role of the funding source

The funders of the study had no role in study design, data collection, data analysis, data interpretation, or writing of the report.

## Results

*T cruzi* was cultured from three clinical studies in Bolivia (appendix 1 p 17). Although samples were cryopreserved, few cells were frozen per tube, and all samples were non-viable after thawing. This non-viability of samples prevented further expansion or isolation of sufficient material for long-read sequencing. Of the 18 originally identified positive cultures, 15 samples had sufficient DNA material for library preparation and were used for Illumina short-read sequencing. We found that the allele frequencies for these samples centred around 1 and 0.5 p, suggesting that these parasite isolates were likely clonal (appendix 1 p 17).

**Figure 1:**
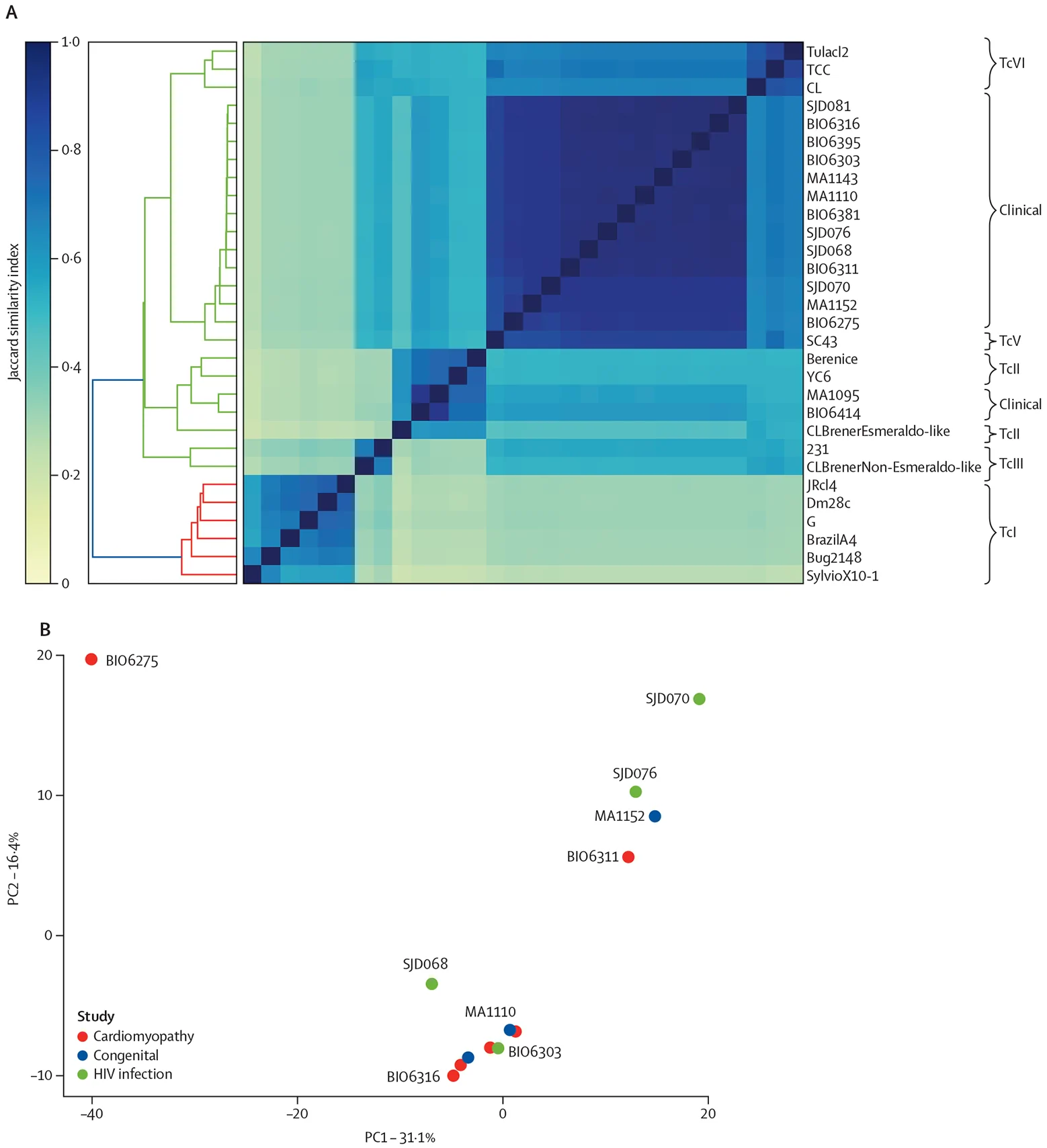
15 clinical isolates from different infection contexts in Bolivia displaying underlying genetic variability (A) Jaccard similarity matrix of k-mer content across assembled genomes. Dark blue indicates high similarity and yellow indicates low similarity in k-mer overlap. Hierarchical clustering of samples based on the Jaccard similarity matrix is shown in the dendrogram. (B) Principal component analysis of allele distances among genes in hybrid genomes.

We estimated the genetic lineage of each isolate by comparing its assembly to reference genomes of known DTUs (figure 1A). Of the 15 isolates, 13 clustered most closely in the k-mer matrix with the hybrid TcV reference genome, and the remaining two clustered with TcII reference genomes, consistent with previous work in the same Bolivian population.^20^ In the absence of a phased genome for *T cruzi* DTU TcV, we mapped raw reads from hybrid strains to the best available reference genome for hybrid lineages (TcVI TCC genome) to assess additional genetic variation. We subsequently identified single nucleotide polymorphisms and performed principal component analysis of allele frequencies to estimate genetic distance among parasites of the same genetic lineage and found genetic structure within isolates of the same lineage, indicating some degree of genetic variation (figure 1B).

In addition to single nucleotide polymorphism-level variation, *T cruzi* undergoes gene copy number variation.^5^ We identified regions of copy number variation across all contigs and samples and found that genomic windows containing MGF members were more likely to show copy number variation (appendix 1 pp 6-7). Despite this copy number variation, the total genome size ranged from 37 Mb to 44 Mb across isolates, which is within the expected range for *T cruzi* (40 Mb).^6^ We further examined heterozygosity in genomic windows across clinically isolated genomes and found regions with variable heterozygosity across all genomes, indicating loss of heterozygosity at some sites, which might represent genomic adaptation in these parasites (appendix 1 pp 6-7).

**Figure 2:**
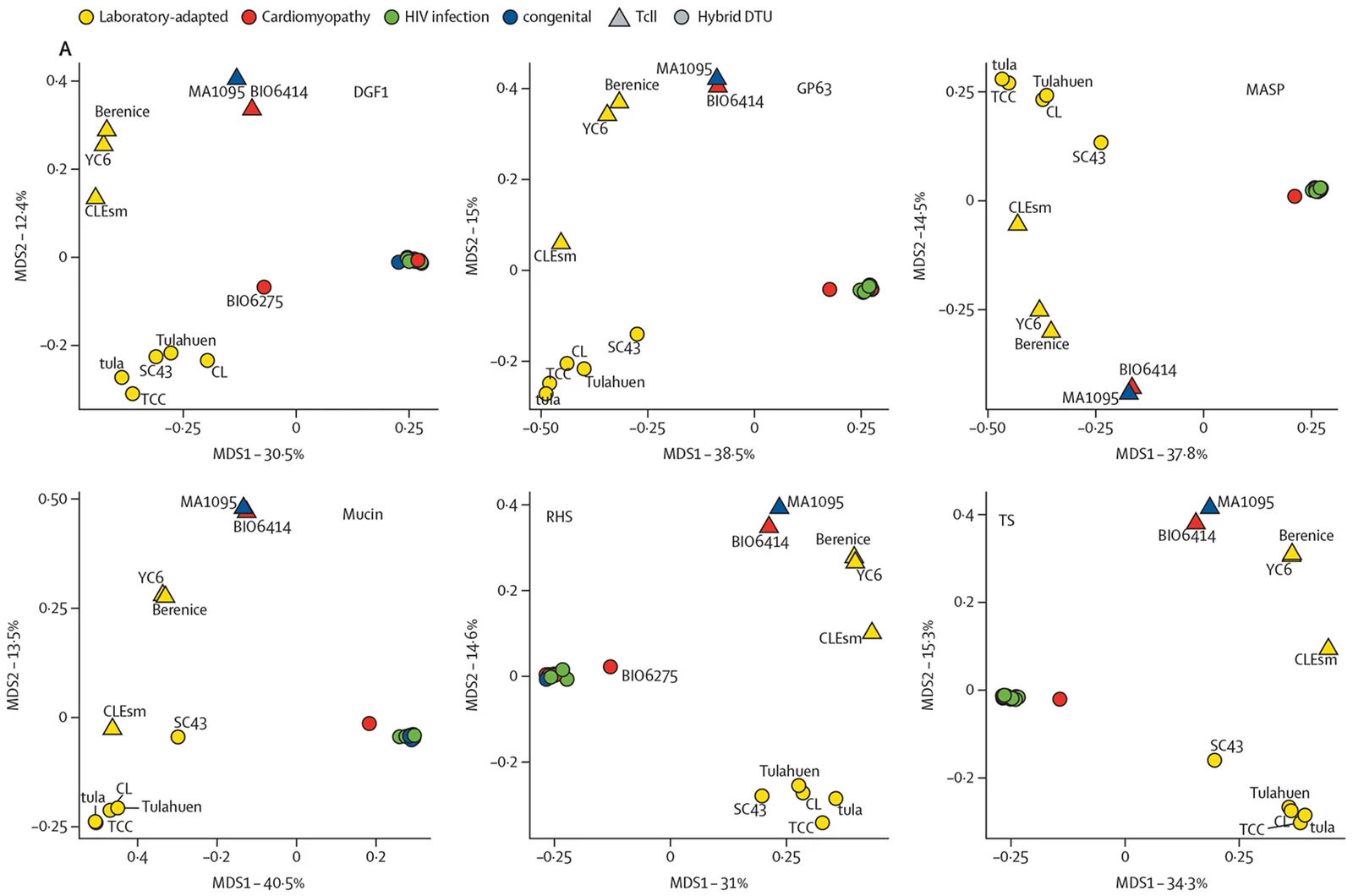

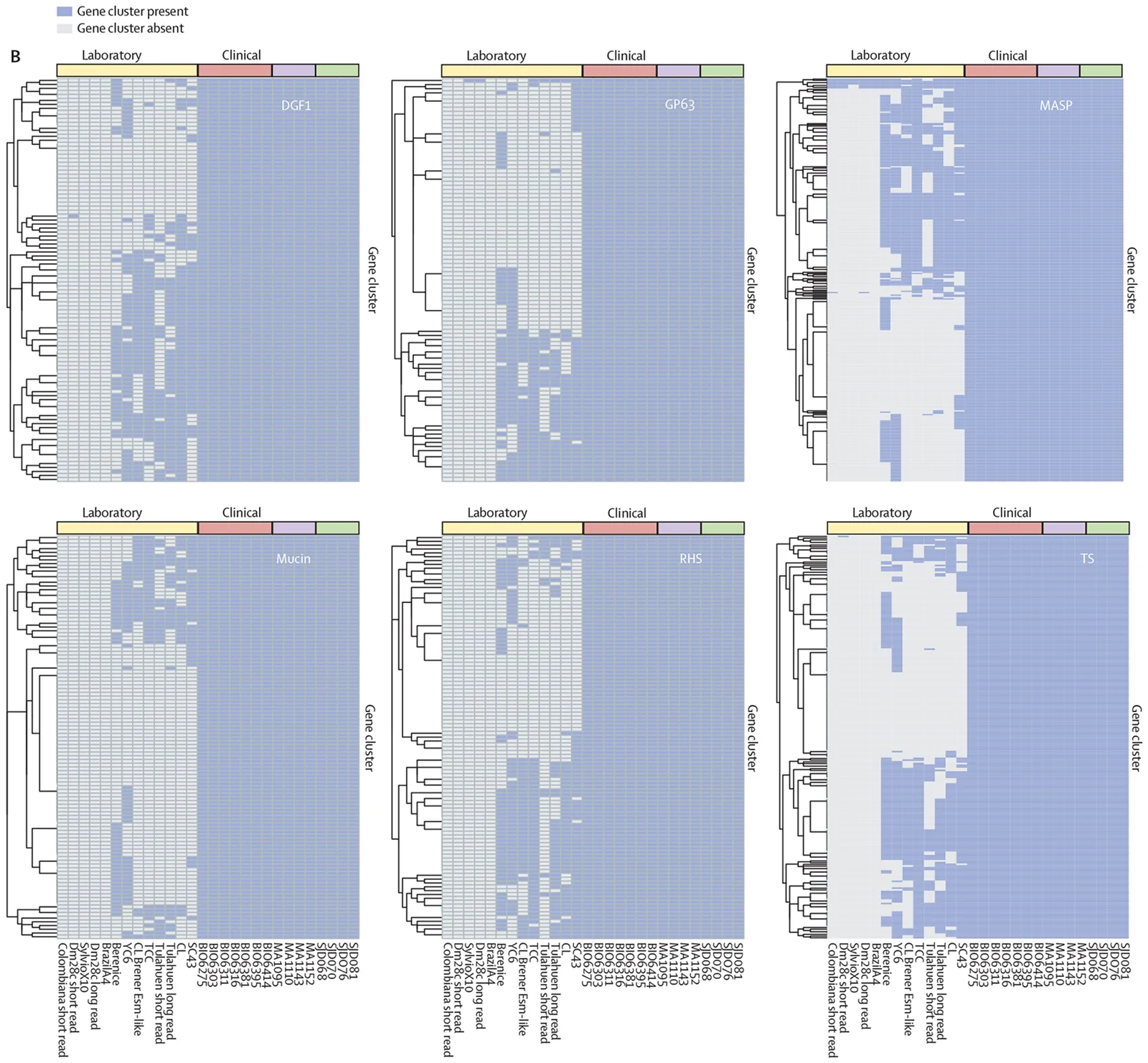

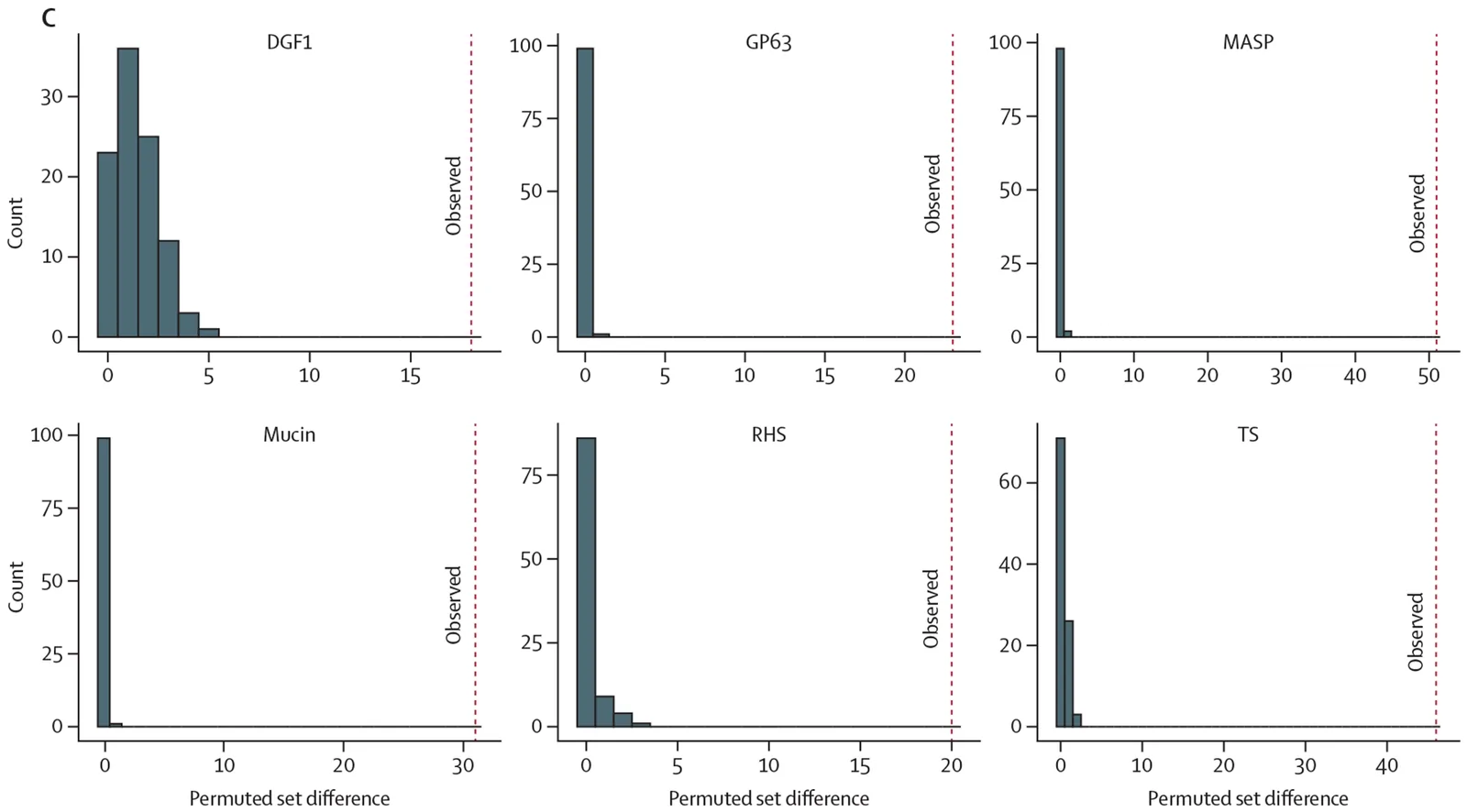
Multigene family repertoires in clinically isolated strains are distinct from those of strains adapted to laboratory culture (A) Multidimensional scaling plots of the Jaccard distances calculated from the presence or absence patterns of gene clusters within each MGF across samples. Point colour indicates study, and point shape indicates parasite genetic type. (B) Presence or absence patterns of gene clusters within each MGF found in all clinical isolates, regardless of DTU. Columns represent strains; yellow bars indicate laboratory-adapted genomes. Blue cells indicate gene clusters present in a genome, and grey cells indicate gene clusters absent from a genome. (C) Histograms of permuted set differences showing the null distribution of the difference between the set of gene clusters present in all clinical isolates and the set present in laboratory-adapted TcII, TcV, and TcVI strains for each MGF. The dotted red line represents the observed set difference between the gene clusters found in all clinical isolates and those found in none of the laboratory-adapted TcII, TcV, and TcVI strains for each MGF. MGF=multigene family. DTU=discrete typing units.

The expansion of MGFs in the *T cruzi* genome is a major source of complexity and has rarely been investigated in clinically isolated strains. As many of these genes are important for infection and likely pathogenesis, the repertoire maintained in laboratory-adapted *T cruzi* strains might differ from that in clinically isolated strains.^11^ To assess MGF repertoire membership in the assembled *T cruzi* genomes, we annotated MGFs in clinical isolate assemblies as previously described; for the laboratory-adapted strains, annotations were generated using previously published long-read and short-read assemblies and in-house generated short-read assemblies.^23,24^ To measure MGF diversity in fragmented short-read assemblies, we assessed MGF gene cluster repertoires across samples. These clusters segregated based on DTU as expected but did not segregate by clinical context (figure 2A). Instead, we observed a clear difference between laboratory-adapted and clinically isolated strains. We identified a subset of gene clusters present in every clinical isolate and absent from all the queried laboratory-adapted strains, regardless of DTU (figure 2B). This observation was tested using permutation of gene clusters across isolate types and was unlikely due to chance (appendix 1 p 19; figure 2C). Our current understanding of *T cruzi* genetic ancestry suggests that these gene clusters, exclusive to a set of clinical isolates that includes both TcII and hybrid strains, are likely present in a common ancestor of both lineages.^3^ We did not identify shared sequence motifs in the translated representative sequences of the clinically conserved gene clusters.

We excluded TcI reference genes from MGF repertoire comparisons because our study did not have any clinically isolated TcI specimens (appendix 1 p 11). To assess whether these findings are generalisable to TcI parasites, we repeated the analysis using previously published whole-genome sequencing data from parasites isolated from Ecuadorian vectors and two Colombian individuals.^25,26^ We did not observe the same separation between clinical isolates and laboratory-adapted strains (appendix 1 p 8). Additionally, we found no overlap between the MGF members shared in our Bolivian clinical isolates and the MGF repertoires of other TcI *T cruzi* field isolates from vectors, suggesting that these genetic lineages have been evolutionarily separated for a long period, such that they no longer share MGF gene clusters.

**Figure 3:**
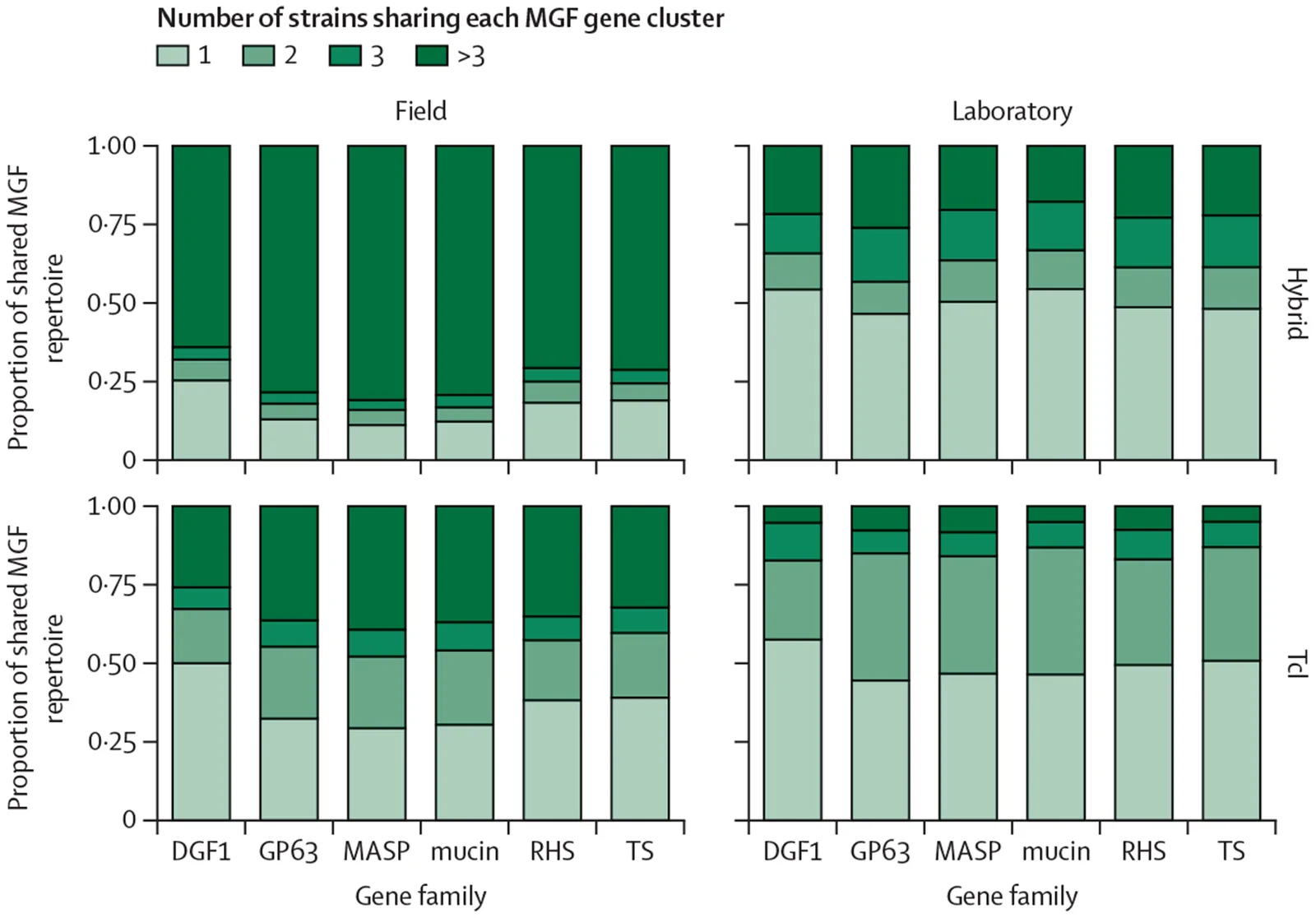
MGF repertoires are more conserved in recent field isolates than in laboratory-adapted strains, regardless of DTU Proportion of MGF repertoire shared across DTUs and isolation recency. Proportional bar plots show, for each MGF, the number of TcI field isolates, laboratory-adapted TcI strains, hybrid field isolates, and hybrid laboratory-adapted strains containing each gene cluster. MGF=multigene family. DTU=discrete typing units.

We observed that MGF gene clusters found in TcI laboratory strains are more likely to occur in one or two laboratory strains, whereas gene clusters found in hybrid DTUs are more likely to occur in two or more strains, suggesting that Tcl parasites have more divergence in MGF repertoires within that lineage compared with that of hybrid strains. This increased divergence in MGF repertoires might explain the absence of clustering observed among TcI strains (appendix 1 p 8). Despite this divergence, MGF repertoires were conserved among field-isolated strains in both hybrid and TcI DTUs, with minimally passaged field isolates showing greater repertoire overlap than laboratory-adapted strains (figure 3). Although we cannot compare exact MGF repertoire sizes across samples due to the limitations of short-read sequencing, these findings suggest some evolutionary pressure for the maintenance of larger MFG repertoires in circulating parasite strains.

## Discussion

In this study, we isolated and sequenced *T cruzi* genomes from a diverse population of individuals living with Chagas disease. To the best of our knowledge, this study provides the first whole-genome sequencing data for clinically isolated hybrid strains and reveals regions of variable copy number across clinical isolates, potential sites of gene conversion, and MGF repertoires that differ from those observed in laboratory-adapted strains. We further identified a set of MGF gene clusters conserved across Bolivian clinical isolates, regardless of parasite genetic ancestry, and lost in laboratory-adapted strains; similarly, we observed a general decrease in MGF repertoire conservation in laboratory-adapted strains compared with minimally passaged parasites, independent of DTU. We compared genomes of recent clinical isolates with those of laboratory-adapted strains and identified features of the parasite genome that might be relevant to clinical infection, highlighting the value of understanding *T cruzi* genomes in field isolates.

Our whole-genome approach enables a holistic investigation of genome diversification in each isolate. Studies in vitro have shown the genomic plasticity of *T cruzi*, and previous work on field isolates has reported genome copy number variation and the possibility of meiotic recombination.^25,26^ In this study, we observed similar plasticity in parasites isolated from human infections. These variable genomic regions highlight genes that might contribute to adaptation in the host environment. We found that genomic regions with increased homozygosity were depleted of MGFs, whereas regions with copy number variation were enriched for MGFs. Thus, these findings suggest a compartmentalisation of genomic content, with signatures of genome diversification enriched in regions encoding MGF members and patterns of genome maintenance enriched in regions encoding core genomic features.

In this study, we were originally interested in identifying differences in parasite genomes associated with specific infection contexts, including variation in parasitaemia, age, and disease severity. These parasites have likely been circulating within each individual for several years, as participants were sampled in an urban setting where the probability of reinfection is low, providing ample time for the parasites to accumulate common mutations associated with the specific infection context. However, we were unable to observe differences in nucleotide diversity, MGF repertoire, or copy number variation specific to clinical infection context. Notably, all samples from this study represent chronic infections, which is potentially a more powerful driver of genomic adaptation than specific infection context, and might therefore explain the genetic similarity among the isolates. Although pregnancy and acute reactivation due to HIV are associated with immunocompromise, this immunocompromise occurs over a relatively short timeframe during which unique genomic changes are unlikely to arise. Our inability to define genotypes associated with specific clinical contexts might also be attributed to the small sample size (as few as four isolates per group), resulting in insufficient power to detect such differences in a whole-genome approach. Nonetheless, regions that we identified as genomically plastic across our isolates might be the focus of future targeted analyses that do not require the intensive strain isolation procedure used in this study.

Although parasite genomes did not cluster genomically based on infection context, we observed an underlying genetic structure, potentially related to other epidemiological factors, most notably the geography of where the participants were initially infected. Because participants were likely infected many years before sampling and the exact timing of infection is unknown, accounting for geographical distribution was nearly impossible in our analyses. A larger study, or one specifically designed to control for geographical variability, might better reveal genomic features associated with specific clinical phenotypes and increase the generalisability of our results.

Although we observed no changes associated with specific clinical contexts of infection, we found marked differences between clinically isolated and laboratory-adapted strains. Our results suggest that MGF repertoires diverge during adaptation to laboratory conditions, with decreased overall conservation of MGF repertoires among laboratory-adapted parasite strains and a subset of MGF genes that are consistently lost in these strains but maintained in Bolivian clinical isolates. Notably, we did not find any of these conserved MGF gene clusters in previously published TcI data, potentially owing to the genetic distance between TcI and TcII or hybrid *T cruzi* strains. We could not determine the repertoire size of MGF members in clinical strains for comparison with laboratory-adapted strains owing to well-characterised issues in assembling multicopy and repetitive regions using short reads.^27^ However, for the MGFs that we assembled and annotated, the number of gene clusters found in all clinically isolated strains and absent from all TcII or hybrid laboratory strains was much higher than would be expected by chance. Although we cannot account for unassembled MGFs, active pressure likely maintains these specific genes in clinically infective strains or eliminates them from laboratory-adapted strains. The maintenance of a conserved antigenic gene repertoire among genetically related strains suggests that common features might be present in conserved MGFs that are essential for infection. Long-read genome assemblies from additional samples might help to elucidate the biological mechanisms driving this conservation.

Besides potential mechanistic implications, this shared repertoire of genes, many of which are potentially immunogenic, might provide a promising avenue for the development of new, targeted multi-antigen diagnostics, which could outperform current diagnostic modalities. Differences in parasite genetics might drive low serological test sensitivities, a major issue in Chagas disease diagnosis, which is primarily performed using serological tests. Some MGF members encode highly immunogenic proteins, and recombinant serological assays often use motifs from these genes as targets. Serological tests have the lowest sensitivity in Mexico and Central America—regions where TcI parasite strains are predominant.^28-30^ Despite within-DTU conservation of MGF repertoires, we observed very little overlap between MGF repertoires of TcI field-isolated parasites and TcII or TcV clinically isolated parasites, potentially explaining the low sensitivity of serological tests that are based on MGFs from type TcV or TcVI strains in regions where TcI strains are predominant. A better understanding of the overlap of MGF repertoires within each DTU might uncover conserved genes within that parasite group, enabling the discovery of new serological targets and the development of more sensitive tests.

A limitation of this study is that we only sequenced parasite strains that could grow in culture. Therefore, additional diversity in clinical isolates that we could not capture is likely. Additionally, some genes that are especially important to clinical infection, potentially impeding parasite survival in culture, could not be studied under these circumstances. Our recovery of only a single parasite clone for each sample might be a result of the bottleneck associated with serial passage in culture. Additionally, the surviving clones from each participant might have genomically adapted to culture during the 50–100 days that the parasite was maintained in culture, although this duration, essentially the minimum required to recover *T cruzi* field isolates, corresponds to very few cell cycles and thus provides limited opportunity for genomic change.^17,25^ Notably, although the samples were isolated from various clinical contexts at larger tertiary hospitals, the patients were likely all infected in the Bolivian Chaco or the inter-Andean valleys of Cochabamba, whereas *T cruzi* is distributed across Latin America. Future studies should evaluate the conservation of these genes in a larger and more geographically diverse sample set.

Finally, we acknowledge that these results are observational, as we could not recover viable parasites to follow up with mechanistic or experimental validation. Future studies will be required to experimentally define the mechanisms underlying our observations. In particular, evolution experiments in vitro could inform the mechanisms driving genome diversification; murine infection models could help to determine the virulence effects of the conserved MGFs we identified.

In Santa Cruz, Bolivia, where these parasites were isolated and where the hybrid TcV strain is predominant, one in five people has Chagas disease.^20^ With advances in technology, the extent of genomic variability that exists within *T cruzi* strains is becoming more apparent. Despite this growing body of knowledge, little is known about specific virulence factors that might contribute to pathogenesis or account for the range of phenotypic outcomes of Chagas disease. Hybrid parasite strains and parasites isolated from clinical contexts have been especially understudied. In this study, we investigated the genomes of hybrid *T cruzi* clinical isolates, which are the predominant strains in the highly endemic southern cone of the Americas. Therefore, the data presented here represent an important first step in showing that parasites isolated from the clinic are genomically distinct from those that have been adapted to culture. Understanding these differences will be essential to developing more sensitive diagnostic tools and identifying new drug targets for this important yet neglected tropical disease.

## Supplementary Material

supplementary appendix 2

supplementary appendix 1

## Data Availability

All cleaned genome sequencing data have been deposited in the Sequence Read Archive under BioProject PRJNA1271783. All codes to generate analysis and figures are available at GitHub.

## Research in context

### Evidence before this study

We searched PubMed for full-text articles in any date range without language restrictions, using the search terms “*Trypanosoma cruzi*” and “genome sequencing”. We only included articles about the organism *Trypanosoma cruzi* and excluded amplicon or multilocus typing, only including articles that performed whole-genome sequencing on clinical samples specifically, without consideration of study bias or study quality. Although several groups performed whole-genome sequencing on *T cruzi* isolated from vectors, only two studies provided whole-genome sequencing data of clinically isolated parasites, all of which were of the parasite genetic type TcI. These studies reported genomic differences between strains isolated from patients with cardiomyopathy and those isolated from patients without symptoms, suggesting that genomic features might be related to clinical outcomes.

### Added value of this study

We generated and analysed the first set of parasite genomes from clinically isolated TcV strains of *T cruzi*, the predominant strain in the highly endemic southern cone of the Americas. We observed genomic heterogeneity within these samples. To our knowledge, for the first time, we describe possible compartmentalisation of diversity-generating and diversity-eliminating genomic changes in the genomes of parasites circulating in human hosts. We compared these genomes with those of laboratory-adapted *T cruzi* strains; our findings revealed key genomic differences between clinically isolated samples and laboratory-adapted parasite strains. Our data show that genomic studies based on parasites adapted to long-term culture in vitro do not fully capture the genomic characteristics of parasites causing human infection. We identified a shared repertoire of variable and immunogenic gene families among clinical isolates that is absent from the genomes of assessed laboratory-adapted strains, indicating a potential role for these genes in establishing or maintaining clinical infection in humans.

### Implications of all the available evidence

The data presented in this study provide evidence for *T cruzi* genomic variability across clinically isolated strains and highlight differences that might be relevant to clinical infection. The shared repertoire of antigenic genes in clinically isolated samples that appear to be lost from laboratory-adapted strains could provide insights into the mechanisms by which *T cruzi* adapts to its human host and inform the development of new serological diagnostic assays, which typically rely on the detection of antibodies against genes within the antigenic and variable multigene families of *T cruzi*. Future work should focus on the genomes of clinically isolated strains, which will better capture genomic variability related to the different clinical phenotypes observed in Chagas disease.
