# Supplementary material for: Genomic diversity of Trypanosoma cruzi clinical isolates: a retrospective analysis
Source: Lancet Microbe. Author manuscript; Available in PMC 2026 Sep 26. (PMC13615562; doi:10.1016/j.lanmic.2026.101446)
Supplement: Supplementary file 1 [file NIHMS2208885-supplement-supplementary_appendix_1.pdf]

THE LANCET
Microbe

Supplementary appendix 1

This appendix formed part of the original submission and has been peer reviewed.
We post it as supplied by the authors.

Supplement to: Hakim JM C, Guarnizo SAG, Duran A, et al. Genomic diversity of *Trypanosoma cruzi* clinical isolates: a retrospective analysis. *Lancet Microbe* 2026. https://doi.org/10.1016/j.lanmic.2026.101446

Contents

Supplemental Figures and Tables 2
- Supplemental Figure 1: 3
- Supplemental Figure 3 5
- Supplemental Figure 4 6
- Supplemental Figure 5 8
- Supplemental Figure 6: 10
- Supplemental Table 1 11
- Supplemental Table 2: 12

Supplemental methods: 13
- Short read whole genome assembly 13
- Reference mapping-based analyses 14

Supplemental Results 16
- Success rates of hemoculture 16
- Assessment of clonality in the isolates 16
- Copy number variation 17
- Regions of heterozygosity loss are depleted of MGF members 18
- Permutation test 18
- Blast search for missing MGFs in lab adapted strain assemblies 19

Supplemental discussion 20
- Double strand repair break as a diversity regulating tool in *T. cruzi* 20

REFRENCES 22

Supplemental Figures and Tables

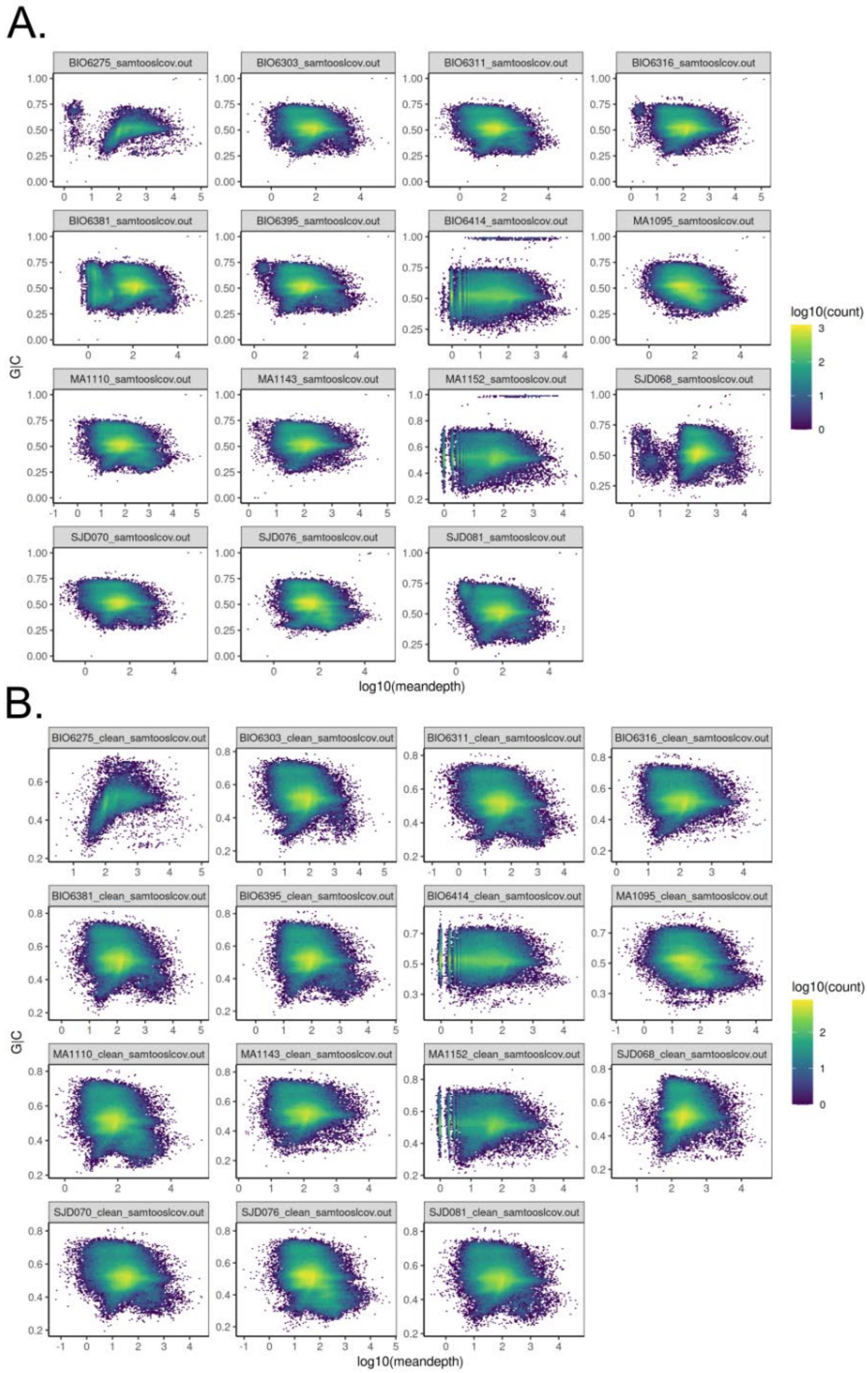

Supplemental Figure 1: 2D histograms of GC percent of each assembled contig and mapping coverage for raw reads to contig for clinical isolate assemblies before (A) and after (B) cleaning contaminating DNA and kDNA demonstrating the removal of contaminating contigs.

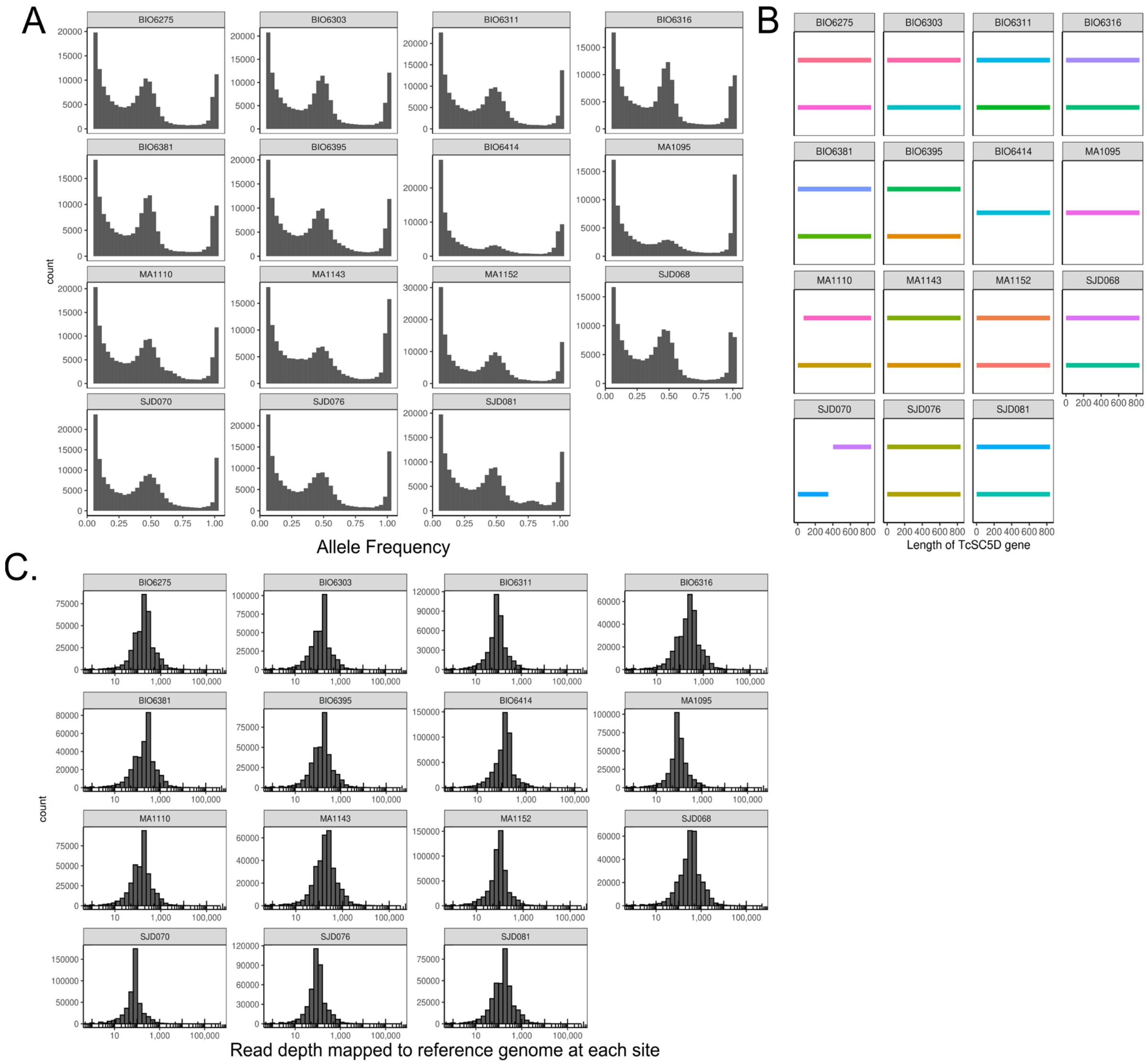

Supplemental Figure 2: A. Distribution of allele frequency for clinically isolated strains against the CL Brener Esmeraldo-like reference genome. Isolates BIO6414 and MA1095 have smaller peaks at 0.5, due to their non-hybrid nature, and lower prevalence of heterozygous alleles. B. Regions of assembled genomes for each strain that map to the putatively single copy TcS5D gene. X axis is the length of the gene, and each colored line is a portion of the assembled gene that maps to the Cl Brener reference. The hybrid strains have two copies, likely corresponding to phased alleles in the assemblies. A complete gene was not assembled for SJD070, though based on mapping coordinates we do not observe more than one copy C. Distribution of read depth for cleaned sequenced reads mapped to the Cl Brener reference genome at each site.

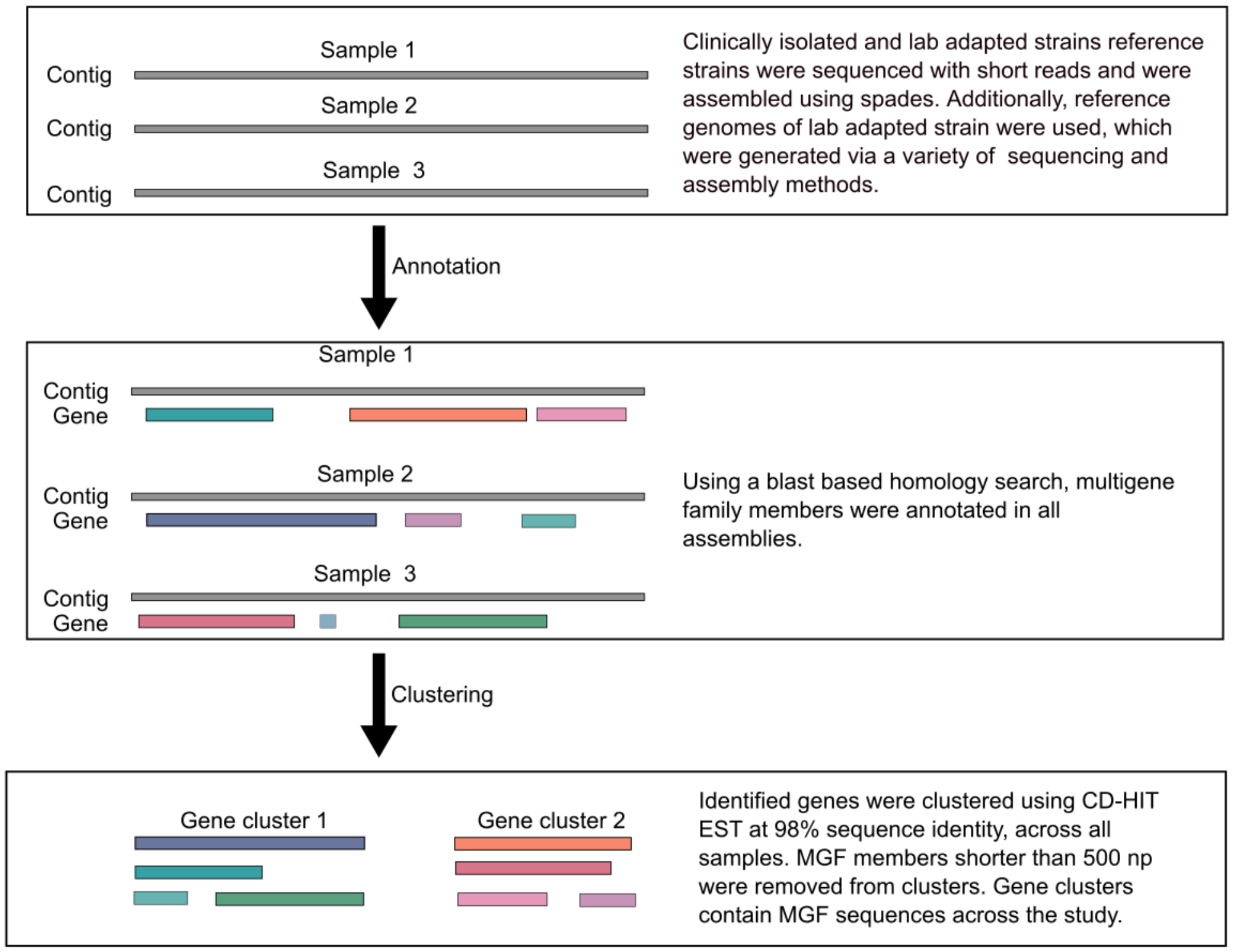

Supplemental Figure 3: Schematic representation of gene clustering strategy for multigene family members

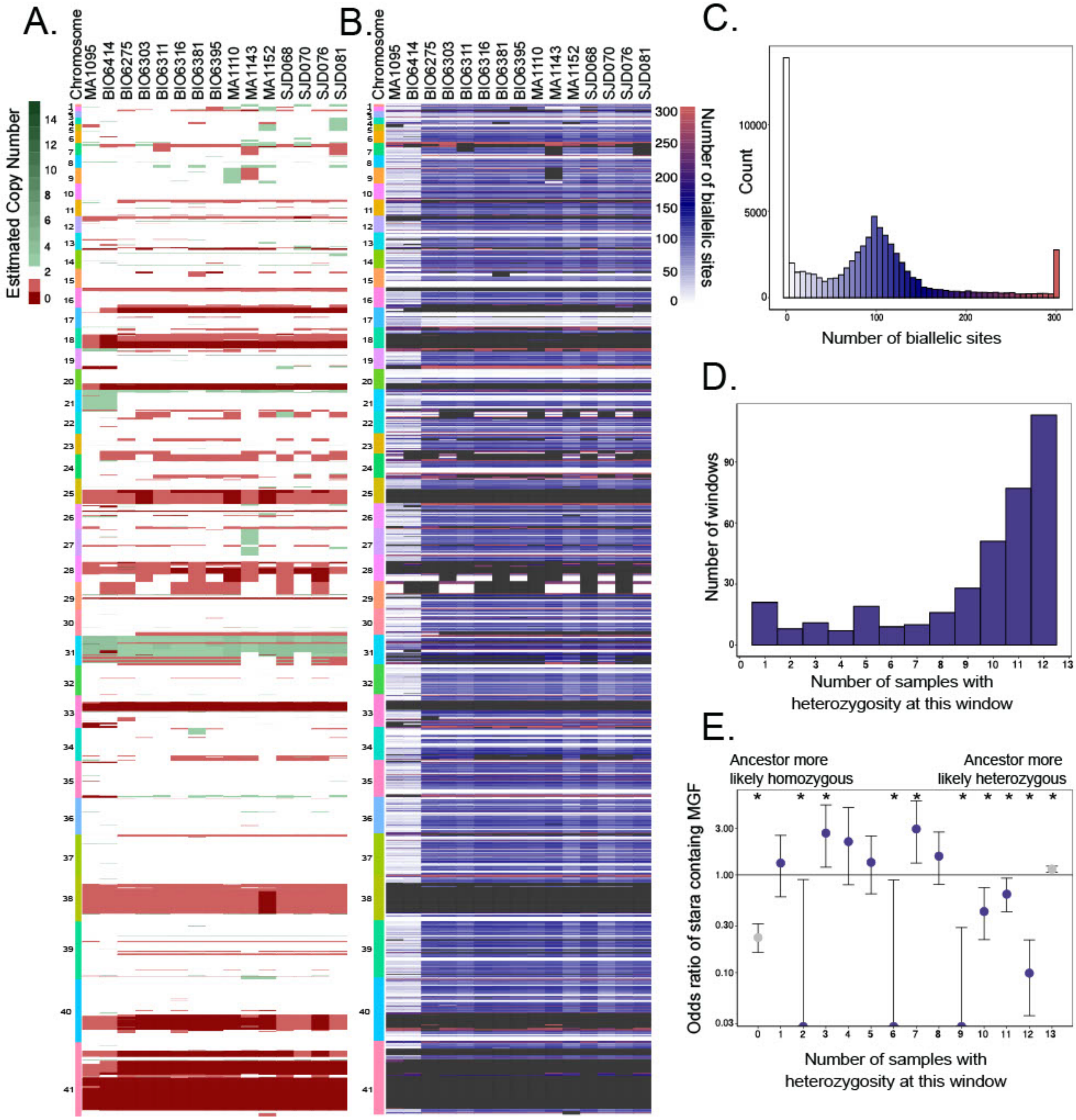

Supplemental Figure 4: **A)** Estimated copy number variation for 5 kb windows in clinically isolated parasite genomes. Each row is a 5kb window colored by loss (red colors) or gain (green) of genomic content at that site. Copy number gains are censored at 15. **B)** Sum of sites in each genome with exactly two alleles in each 5kb window. White indicates regions with

very few biallelic sites, indicating homozygosity, while blue then red indicates an increase of biallelic sites. Windows are censored at biallelic sites = 300. Grey boxes are masked regions that have less than two estimated copies, which would by definition have one copy of the allele. **C)** Histogram showing the distribution windows with a given number of biallelic sites. The heterozygous peak is largely at 100 biallelic sites per 5kb, or 2%, and therefore a minimum threshold of 1% of biallelic sites is set for calling a window heterozygous. The colors correspond to figure 2B. **D)** Heterozygosity concordance among TcV samples. For each window, the number of samples where that window is largely heterozygous (greater than 1% of sites in the window are biallelic) was calculated. The number of windows that are heterozygous in one out of 13 total samples TcV samples, up to 12 out of 13 TcV samples is shown. The majority of windows had perfectly concordant heterozygosity (n = 1035) or concordant homozygosity (n = 2985). The sites without perfect concordance in heterozygosity in all TcV samples are marked as possible gene conversion events, in blue. **E.** The odds ratio of a window in each stratum defined in figure 2D to have MGF members. Windows with 2, 6 and 9 samples sharing heterozygosity had zero observed MGFs and thus the CI reaches 0. Asterisks indicate ORs that are statistically significant after correction via Benjamini-Hochberg Procedure. ORs and CIs are available in Supplemental Table 3.

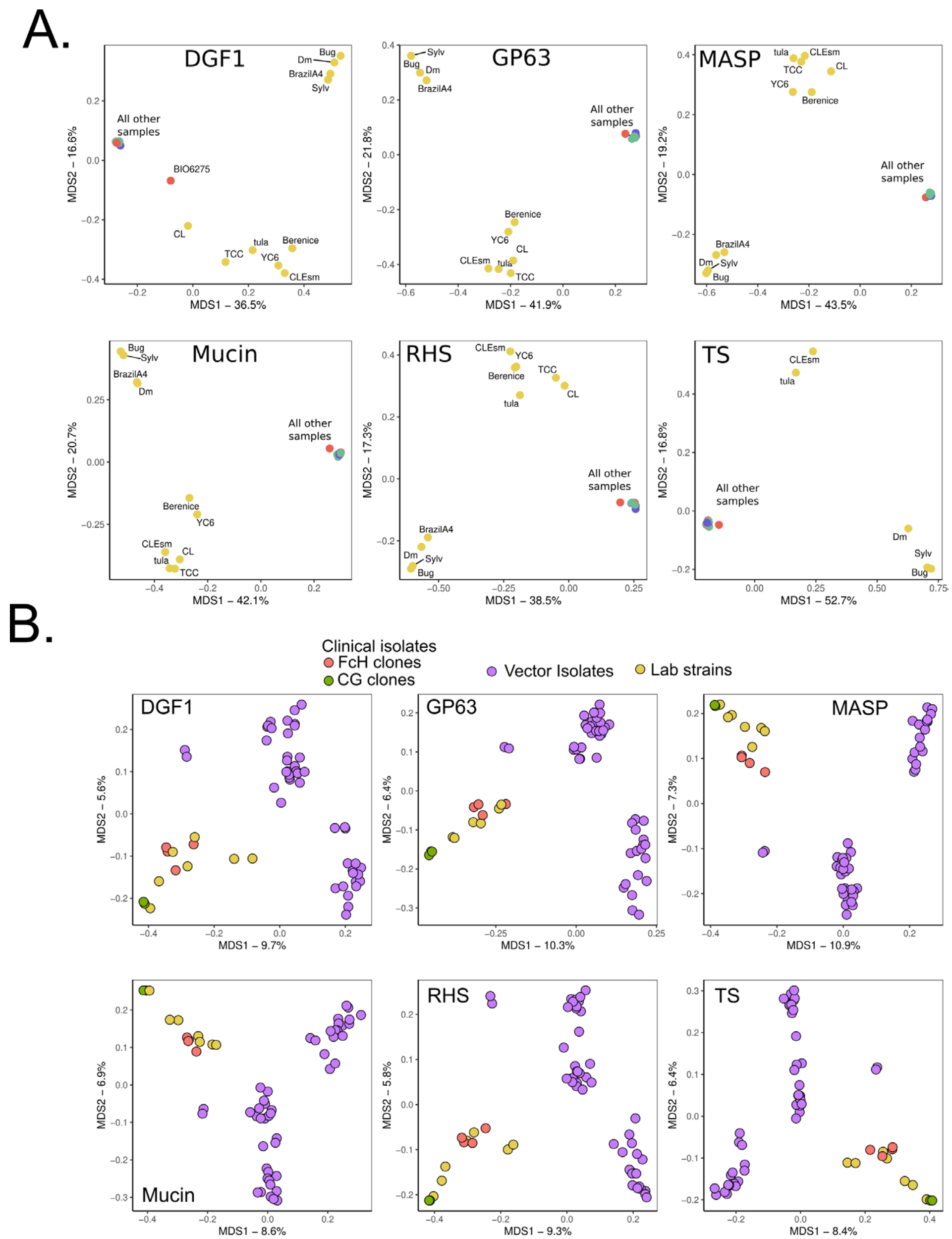

Supplemental Figure 5: A) MGF repertoire separation including Tc1 lab reference strains. DTU separation is more pronounced but note that due to the lack of clinically isolated specimens in the current study some separation may be largely DTU driven. B) MGF repertoire separation of TcI parasites alone, including Colombian clinically isolated parasites from Talavera-Lopez et al (FcH and CG, red and green respectively) and vector isolates from Schwabl et al. (purple). FcH and CG are two individuals for which there were four and two parasite genomes cloned from the patient samples

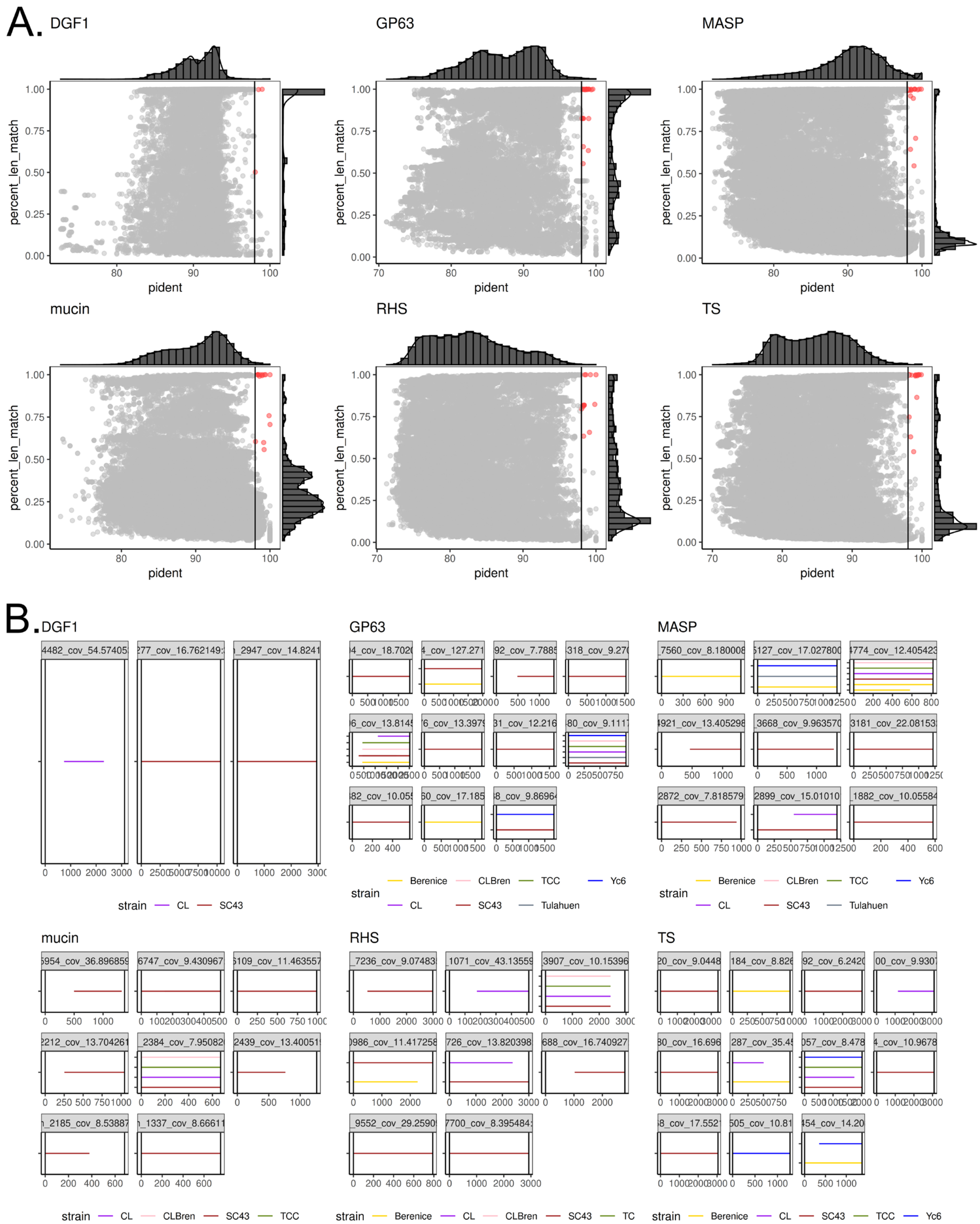
A.
DGF1
GP63
MASP
mucin
RHS
TS
percent_len_match
pident
B.
DGF1
GP63
MASP
mucin
RHS
TS
strain
CL
SC43
Berenice
CLBren
TCC
Yc6
Tulahuen
TC

Supplemental Figure 6: Confirming the loss of shared MGFs among lab adapted strains by BLASTN search of lost representative sequences within whole genomes of TcII, TcV and TcVI strains. **A)** Plot of all BLASTN hits in reference genomes for each lost MGF encoding gene, across all gene families. X axis represents the percent identity of the BLASTN hit, and Y axis represents the percent of the total MGF that is covered by the BLASTN homology found in the reference genome. The vertical line is at 98% BLASTN sequence identity. Points colored in red are above 98% sequence identity and at least 50% query coverage and are the sequences that were selected for the second panel. **B)** Each MGF member that passes the above thresholds. Each gene is a facet, colored by the reference genome the gene was found in. X axis is the length of the gene, with black bars demarcating that start and end, and line segments of each reference genome representing the position where the hit occurred. These are the genes that were removed from the set of shared clinical MGF members that were lost in the lab adapted strains.

**Sample ID**	**numreads_R1**	**numreads_R2**	**HE**	**Haploid Genome length**	**Assembly len**	**# Contigs**	**Average Contig Length**	**n50**	**Single (%)**	**Duplicated (%)**	**Fragmented (%)**	**Incomplete (%)**	**Missing (%)**
BIO6275	3463629	3512846	2.49455	40818222	46568153	28140	1654	3943	77.69	7.69	13.08	0.77	0.77
BIO6303	2452701	2432325	2.34576	37324623	69848654	126469	552	1813	51.53	16.92	27.69	0.77	3.08
BIO6311	1615176	1582604	2.48213	40271855	72304979	151300	477	1512	62.30	15.38	20.77	0.00	1.54
BIO6316	3971510	3949092	2.41809	38018721	70828901	128911	549	1862	44.61	16.92	34.62	0.77	3.08
BIO6381	4095168	4101251	2.36717	37091970	71230598	131032	543	1763	46.92	16.15	33.08	0.00	3.85
BIO6395	2961480	3002744	2.51745	40035111	71052600	135798	523	1780	57.69	16.15	23.85	0.77	1.54
BIO6414	1793955	1774465	1.0475	37618289	57681790	156709	368	770	100	0.00	0.00	0.00	0.00
MA1095	1804518	1798788	0.984298	42523145	58393558	174822	334	627	100	0.00	0.00	0.00	0.00
MA1110	2825007	2820456	2.44574	40467366	73701203	153886	478	1561	48.46	15.38	31.54	0.77	3.85
MA1143	3421261	3419717	2.48146	39985326	69584163	122999	565	2003	44.61	17.69	33.85	0.77	3.08
MA1152	1439860	1451530	2.52186	39394166	62347121	104101	598	2061	63.07692	10.77	24.62	0.00	1.54
SJD068	6413595	6601890	2.54625	38631308	70882885	137934	513	1523	55.38462	11.54	29.23	1.54	2.31
SJD070	1187680	1168239	2.58895	39728401	67533274	136496	494	1527	73.07692	8.46	16.92	0.00	1.54
SJD076	2000918	2022794	2.48158	44142051	79778165	196128	406	1244	57.69231	11.54	27.69	0.77	2.31
SJD081	2743003	2707380	2.47528	39546644	70022023	127496	549	1854	49.23077	16.15	30.77	0.77	3.08

Supplemental Table 1: Genome assembly statistics.

Population	Feature	Aggregated metric
Peripartum (N=4)	Mean Age (SD)	28.20(2.18)
	Percent Men (N)	0% (0)
	Parity mean count (SD)	2.75 (1.92)
	Percent with known exposure (N)	25% (1)
	Percent transmitted to infant (N)	25% (1)
	Percent Hispanic (N)	100% (4)
Persons with HIV (N=4)	Mean Age	47.75 (14.11)
	Percent Men (N)	100% (4)
	Mean CD4 Count (SD)	278.3 (79.27)
	HIV Load in mean copies/ml (SD)	33,364.66 (36,026.11)
	Percent positive Micromethod (N)	75% (3)
	Mean *T. cruzi* parasite per mL (SD)	1,449,762.25 (2,571,622.078)
	Percent Hispanic (N)	100% (4)
Participants in study of Chagas cardiomyopathy (N=6)	Mean Age (SD)	56 (12.79)
	Percent men (N)	50% (3)
	Percent with ECG abnormalities (N)	50 % (3)
	Percent Hispanic (N)	100% (6)

Supplemental Table 2: Aggregated characteristics of study populations

Supplemental methods:

Short read whole genome assembly

Raw reads were assembled using SPAdes (v4.2.0) with kmer lengths of k = 21,33,55,77,99, and 127. (1) To eliminate contaminating DNA from other organisms, a search for known *T. cruzi* sequences using BLASTN (v 2.17.0) to query all assembled contigs against a concatenated library of the following high quality reference *T. cruzi* genomes representing different parasite genetic types: CL Brener Esmeraldo-like, CL Brener Non-Esmeraldo-like, G, BrazilA4, JRcl4, TCC, Tulacl2, Tulahuen, SlyvioX10, YC6, Bug2148, 231, Berenice, Dm28c and CL(2–10). Raw reads were mapped to the pre and post-cleaned assemblies using bowtie2 (v 2.5.4) (11). Removal of non-*T. cruzi* contaminants was visualized in 2D histogram plots of GC content vs median contig coverages (**Supplemental Figure 1**). These non-*T. cruzi* sequences were extracted using seqkit (v2.12.0))and classified using bbmap sendsketch (v38.18)(12,13). Contaminating contigs most often matched bacterial sequences, the most common of which were Dietzia maris, Mycobacterium tuberculosis, and Salmonella enterica. The full list of these contaminants is available on the Github. Raw fastq files were cleaned of contaminating sequencing reads using SAMtools (v1.3.1) (14). The cleaned assemblies and fastqs were used in all further analyses. In addition to the clinical specimens, the following lab adapted strains were sequenced in house and processed in the same way to improve comparisons: Dm28cCas9, Tulahuen, and Colombiana.

Heterozygosity was assessed in clinical isolates using Jellyfish (v 2.3.0) to generate kmer histograms, with a kmer length of 21, then Genomescope2.0 (v2.0) using default parameters (15,16). Assembly statistics were assessed using Assembly-stats, BUSCO scores were calculated

using Complasm (v0.2.7) (17). All assembly statistics and sequencing statistics are reported in **Supplemental Table 1**. Genetic distances between genome assemblies and known strains were calculated and visualized with Sourmash (v4.7.0) (18).

Multigene family members were annotated from clinical isolate assemblies, short read assembled lab strains, and previously generated reference genomes, using the same methodology previously described (8,10). The identified genes were clustered using CD-hit at 98% sequence identity using a word size of 9. After clustering, genes within a cluster shorter than 500 base pairs were removed, to increase specificity and to avoid erroneous clustering of fragmented assemblies. A schematic representation of this approach is in **Supplemental Figure 2.**

BLASTN was used to search for MGFs assembled from clinical isolates that were putatively lost from lab adapted strains. Using the representative sequence from the gene cluster as the subject queried against TcII and hybrid reference strains, all MGFs members that matched an MGR sequence with at least 98% sequence similarity and 50% query coverage to were considered present in the lab strain.

Reference mapping-based analyses

Single nucleotide polymorphisms (SNP) and copy number analysis were performed by mapping each clinical isolate to Cl Brener reference genome (6). All mapping was done using Bowtie2 default parameters. FreeBayes was used to detect SNPs in clinical isolates against the CL Brener Esmeraldo-like reference genome using all default parameters, except for the parameters -F 0.01 -C 1 --pooled-continuous (19). Allele frequency histograms were generated using these SNPs to assess complex infection, excluding sites with coverage less than 10. Read depth at each site was visualized in **supplemental figure 3C.**

Additional genetic distance among hybrid clinical isolates was assessed using the SNP frequency of clinical isolates against the TCC reference genome using InStrain, again excluding sites with coverage less than 10 (20). InStrain SNP data was also generated compared to the Cl Brener Esmeraldo -like reference genome and was used to estimate levels of heterozygosity in 5kb genomic windows, excluding sites with more than 2 alleles and coverage less than 10.

Copy number variation was assessed as previously described(21). Raw reads of each sample were mapped against the CL Brener Esmeraldo -like reference, and Control-FREEC was used to estimate copy number of 5 kilobase windows across the reference genomes(22). Control-FREEC measures the read depth in genomic windows and normalizes the count to GC percent to estimate copy number. To rank genomic windows in order of copy number variability, we calculated for each window the median absolute deviation of normalized read depth coverage at the same site in every sample. We used a Fisher's test to assess the significance of multigene family (MGF) enrichment in the top 5th percentile of variability.

We defined heterozygous genomic regions by counting the number of bases in the genome that contain 2 alleles within a 5kb window. Windows with more biallelic sites have higher heterozygosity, and those with more monoallelic sites have higher homozygosity.

To more closely evaluate potential adaptation in these isolates, we analyzed genetic windows in the TcV clinically isolated genomes that vary in their level of heterozygosity. Given the shared lineage of these parasites, this discordance of heterozygosity must have arisen after these strains diverged from a common ancestor. Discordant windows may have either started out homozygous, later becoming heterozygous by random mutation in only one allele or by homologous recombination with another non-allelic locus to generate additional diversity, or alternatively, started out heterozygous and became homozygous by allelic homozygous

recombination, using the alternate allele as a template. We therefore used the number of samples with shared heterozygosity as a proxy for likelihood that a common ancestor originally had heterozygosity in that window.

Supplemental Results

Success rates of hemoculture

Hemoculture was attempted from 147 patients with confirmed positive *T. cruzi* serology yielding a total of 18 positive hemocultures, resulting in a disaggregated success rate of 5.61% (5/89) from peripartum mothers, 19.57% (9/46) from chronically infected patients, and 33.34% (4/12) from participants with end stage AIDS. The success rate of these isolates is unlikely to be related to the initial parasitemia in each blood sample because many different technicians isolated samples over the year of collection, and there are therefore many possible technical factors that could have contributed to success rate. Most of the isolated parasites belonged to TcV DTU; it is possible that something about the TcV parasites make them more amenable to successful hemoculture. However, we believe this is mostly a function of the epidemiology of regional parasite strains, as previously published work in the same population has revealed a preponderance of TcV/TcVI parasites (23).

Assessment of clonality in the isolates

T. cruzi infection is often multiclonal, especially in immunosuppressed individuals, such as pregnant people and people living with HIV (24–26). To computationally assess the clonality of each sample, we mapped the raw reads of each isolate against the CL Brener Esmeraldo-like reference genome. We observed allele frequencies that centered around 0, 1 and 0.5 in all but one

of the sequenced samples, indicating the presence of a single diploid organism (**Supplemental Figure 3A).** One sample, SJD081, has a minority peak at allele frequency 0.75, though the accompanying peak at 0.25 which would be evidence of a minority clone is impossible to detect.

In addition to estimating clonality based on reference mapping, we also searched using BLASTN for copies of the single copy TcS5D gene in *de novo* assemblies of each clinical isolate (27). In each sample, the gene was present in one or two copies. We can detect heterozygous copies, as demonstrated by the gene's resolution in the TcV isolates, and the single copy resolved in the TcII, non-hybrid isolates. (**Supplemental Figure 3B**). Based on these results, we conclude that, although the original patients may have had polyclonal infections, these clinical samples each represent a single parasite clone.

Copy number variation

Some genomic regions, such as those that map to Chr31, have increased copy in all clinical samples, which has been previously observed in field isolates and may represent a universal genomic feature of field isolated *T. cruzi* (12,33). Of particular interest are sites that have variable copy number in multiple clinical isolates, because they may represent recent or specific adaptations to the clinical infection context; for example, some of the genomic information encoded in chromosome 9 with elevated copy number in the MA110 genome shows decreased copy number in the MA1143 genome. In other work, sites with variable copy number are often enriched for multi-copy genes including *T. cruzi's* highly antigenic multigene family members such as mucins and trans-sialidases (10,11,34). We found that the most variable window is significantly more likely to be enriched with MGF members when compared to the background distribution of the whole genome (OR: 3.29 for presence of MGF genes in high CNV variance windows, CI 3.26-3.34). This observation is consistent with other analyses; in

long read genome assemblies, MGFs have variable repertoire sizes in different *T. cruzi* strains [7,10,38].

Variance in copy number for specific genomic regions raises the question of overall genomic content, both in terms of haploid genome length and proportion of the genome that contains heterozygous alleles. Despite the copy number variation observed in figure 2, the total genomic material remains similar across all isolates (37Mb – 44Mb), around the expected range for *T. cruzi* (40 Mb(37)). As expected, the two non-hybrid TcII samples, MA1095 and BIO6414, have lower heterozygosity (0.98% and 1.04%), while the hybrid TcV samples have high heterozygosity (2.35% - 2.59%) **(Supplemental Table 1)**.

Regions of heterozygosity loss are depleted of MGF members

We see that regions that are heterozygous in most samples, and thus likely ancestrally heterozygous before conversion to homozygosity in a subset of samples, are highly depleted of MGFs compared to the background distribution of MGF genes in the genome as a whole (windows where 9, 10, 11 and 12 out of 13 samples are heterozygous at that window, OR = 0 (0-0.20), 0.37(0.29-0.62), 0.50 (0.33-0.71), 0.21 (0.12-0.33)) (**supplemental Fig 4D**). In contrast, we do not observe consistent depletion of MGFs in regions where the majority of sites are homozygous and likely ancestrally homozygous. These observations suggest that recombination is being used both as a mechanism to increase diversity in MGF-containing genomic regions via copy number variation and to eliminate or limit diversity via homozygosity-generating gene conversion in important core genomic features.

Permutation test

We found a subset of gene clusters found in every clinical isolate that was also absent from all the queried lab adapted strains, regardless of DTU. To assess the statistical significance of this observation, and to confirm it was not an artifact of the number of gene clusters we were able to assemble and annotate in each sample type, we permuted the gene clusters found in clinical isolates and the genetically related type TcII, TcV and TcVI lab adapted reference strains to estimate the number of gene clusters that we would expect to be present in all clinical isolates and no lab strains by chance. Across all gene families, the observed frequency of unique gene clusters for each MGF was highly significant as calculated by z score (zscore of DGF1 = 12.17, GP63 = 199.9, MASP =333.38, mucin = 279.9, RHS = 28.7, TS = 74.93, all p values by the z statistic 0.00001).

Blast search for missing MGFs in lab adapted strain assemblies

To more rigorously determine whether these MGFs were truly unique to clinical isolates, we used BLASTN to search for the representative sequence from each gene cluster (the longest sequence within each cluster) within the lab adapted strain's genomes (**Supplemental Figure fig 6A and 6B**). After this filtering out MGFs present in any lab strain (>98% sequence similarity and 50% query coverage), we identified between 15 and 47 genes per multigene family that were unique to clinical isolates and absent from all laboratory strains (DGF1= 15, GP63 = 20, mucin = 28, RHS = 16, Tran-sialidase = 40, MASP = 47).

Supplemental discussion

Double strand repair break as a diversity regulating tool in *T. cruzi*

We found a compartmentalization of patterns of recombination, where genomic regions enriched of MGFs are more likely to be in copy number variable region and regions depleted of MGFs are more likely to be in regions with loss of heterozygosity. Recombination following double stranded DNA breaks is a possible mechanism for both the generation and elimination of diversity that we observe. Antigenic gene copy number may increase over time by gene drift, increasing the potential genomic repertoire for these diverse genes. Because *T. cruzi* is thought to lack transcriptional control, gene dosage could also be a genomic adaptation resulting in phenotypic changes(21). Regions with variable copy number in clinical isolates, including those containing non-MGF genes, may be specifically important to clinical infection, as they may be subject to genomic changes that are known to occur as a result of adaptation to different culture environments at relatively rapid speeds (21). These observations may help to explain how such a genetically plastic organism maintains genetic integrity in core genomic regions**.**

Because *T. cruzi* lacks non-homologous end joining, DNA double strand break repair in trypanosomes occurs via either homologous recombination or microhomology mediated repair (28). In higher order eukaryotes, the template used for homologous recombination is most often the alternate allele on the homologous chromosome, leading to gene conversion that eliminates allelic diversity by removing heterozygous alleles from an individual and a population (29). We observe patterns consistent with this mechanism in regions depleted of MGF members. Increased copy number regions, in contrast, are likely to arise from non-allelic recombination, where unequal crossing over occurs. These regions of unequal crossing over may be templated by highly repetitive transposable elements. We and others have previously observed that MGF

members are closer in linear genome space to transposable elements than other gene groups(8,10,26,30–32). The compartmentalization of diversity-generating and diversity-eliminating recombination may be driven by the increased rate of non-allelic recombination near MGFs, given the abundance of potential recombination templates, either from other MGF members or from transposable elements proximal to MGFs, or could be a result of negative selection against deleterious diversity in core genomic regions. Both possibilities are likely to contribute to the observed compartmentalization

REFRENCES

1. Bankevich A, Nurk S, Antipov D, Gurevich AA, Dvorkin M, Kulikov AS, et al. SPAdes: a new genome assembly algorithm and its applications to single-cell sequencing. J Comput Biol J Comput Mol Cell Biol. 2012 May;19(5):455–77. doi:10.1089/cmb.2012.0021 PubMed PMID: 22506599; PubMed Central PMCID: PMC3342519.

2. Baptista RP, Reis-Cunha JL, DeBarry JD, Chiari E, Kissinger JC, Bartholomeu DC, et al. Assembly of highly repetitive genomes using short reads: the genome of discrete typing unit III Trypanosoma cruzi strain 231. Microb Genomics. 2018 Apr;4(4):e000156. doi:10.1099/mgen.0.000156 PubMed PMID: 29442617; PubMed Central PMCID: PMC5989580.

3. Berná L, Rodriguez M, Chiribao ML, Parodi-Talice A, Pita S, Rijo G, et al. Expanding an expanded genome: long-read sequencing of Trypanosoma cruzi. Microb Genomics. 4(5):e000177. doi:10.1099/mgen.0.000177

4. Bradwell KR, Koparde VN, Matveyev AV, Serrano MG, Alves JMP, Parikh H, et al. Genomic comparison of Trypanosoma conorhini and Trypanosoma rangeli to Trypanosoma cruzi strains of high and low virulence. BMC Genomics. 2018 Oct 24;19(1):770. doi:10.1186/s12864-018-5112-0

5. Callejas-Hernández F, Gironès N, Fresno M. Genome Sequence of Trypanosoma cruzi Strain Bug2148. Genome Announc. 2018 Jan 18;6(3):e01497-17. doi:10.1128/genomeA.01497-17 PubMed PMID: 29348355; PubMed Central PMCID: PMC5773740.

6. El-Sayed NM, Myler PJ, Bartholomeu DC, Nilsson D, Aggarwal G, Tran AN, et al. The Genome Sequence of Trypanosoma cruzi, Etiologic Agent of Chagas Disease. Science. 2005 Jul 15;309(5733):409–15. doi:10.1126/science.1112631

7. Franzén O, Ochaya S, Sherwood E, Lewis MD, Llewellyn MS, Miles MA, et al. Shotgun sequencing analysis of Trypanosoma cruzi I Sylvio X10/1 and comparison with T. cruzi VI CL Brener. PLoS Negl Trop Dis. 2011 Mar 8;5(3):e984. doi:10.1371/journal.pntd.0000984 PubMed PMID: 21408126; PubMed Central PMCID: PMC3050914.

8. Hakim JMC, Gutierrez Guarnizo SA, Málaga Machaca E, Gilman RH, Mugnier MR. Whole-genome assembly of a hybrid Trypanosoma cruzi strain assembled with Nanopore sequencing alone. G3 GenesGenomesGenetics. 2024 Jun 1;14(6):jkae076. doi:10.1093/g3journal/jkae076

9. Lewis MD, Llewellyn MS, Gaunt MW, Yeo M, Carrasco HJ, Miles MA. Flow cytometric analysis and microsatellite genotyping reveal extensive DNA content variation in Trypanosoma

cruzi populations and expose contrasts between natural and experimental hybrids. Int J Parasitol. 2009 Oct;39(12):1305–17. doi:10.1016/j.ijpara.2009.04.001 PubMed PMID: 19393242; PubMed Central PMCID: PMC2731025.

10. Wang W, Peng D, Baptista RP, Li Y, Kissinger JC, Tarleton RL. Strain-specific genome evolution in Trypanosoma cruzi, the agent of Chagas disease. PLOS Pathog. 2021 Jan 28;17(1):e1009254. doi:10.1371/journal.ppat.1009254

11. Langmead B, Salzberg SL. Fast gapped-read alignment with Bowtie 2. Nat Methods. 2012 Mar 4;9(4):357–9. doi:10.1038/nmeth.1923 PubMed PMID: 22388286; PubMed Central PMCID: PMC3322381.

12. SourceForge [Internet]. 2025 [cited 2025 May 23]. BBMap. Available from: https://sourceforge.net/projects/bbmap/

13. Shen W, Sipos B, Zhao L. SeqKit2: A Swiss army knife for sequence and alignment processing. iMeta. 2024;3(3):e191. doi:10.1002/imt2.191

14. Danecek P, Bonfield JK, Liddle J, Marshall J, Ohan V, Pollard MO, et al. Twelve years of SAMtools and BCFtools. GigaScience. 2021 Feb 1;10(2):giab008. doi:10.1093/gigascience/giab008

15. Marçais G, Kingsford C. A fast, lock-free approach for efficient parallel counting of occurrences of k-mers. Bioinformatics. 2011 Mar 15;27(6):764–70. doi:10.1093/bioinformatics/btr011

16. Ranallo-Benavidez TR, Jaron KS, Schatz MC. GenomeScope 2.0 and Smudgeplot for reference-free profiling of polyploid genomes. Nat Commun. 2020 Mar 18;11(1):1. doi:10.1038/s41467-020-14998-3

17. Huang N, Li H. miniBUSCO: a faster and more accurate reimplementation of BUSCO [preprint] [Internet]. Genomics; 2023 Jun [cited 2023 Jun 29]. Report No. Available from: http://biorxiv.org/lookup/doi/10.1101/2023.06.03.543588 doi:10.1101/2023.06.03.543588

18. Brown CT, Irber L. sourmash: a library for MinHash sketching of DNA. J Open Source Softw. 2016 Sep 14;1(5):27. doi:10.21105/joss.00027

19. Garrison E, Marth G. Haplotype-based variant detection from short-read sequencing [Internet]. arXiv; 2012 [cited 2025 May 9]. Available from: http://arxiv.org/abs/1207.3907 doi:10.48550/arXiv.1207.3907

20. Olm MR, Crits-Christoph A, Bouma-Gregson K, Firek B, Morowitz MJ, Banfield JF. InStrain enables population genomic analysis from metagenomic data and sensitive detection of shared microbial strains. Nat Biotechnol. 2021 Jun;39(6):727–36. doi:10.1038/s41587-020-00797-0 PubMed PMID: 33462508; PubMed Central PMCID: PMC9223867.

21. Matos GM, Lewis MD, Talavera-López C, Yeo M, Grisard EC, Messenger LA, et al. Microevolution of Trypanosoma cruzi reveals hybridization and clonal mechanisms driving rapid genome diversification. Claessens A, van der Meer JW, Lukes J, Agüero F, editors. eLife. 2022 May 10;11:e75237. doi:10.7554/eLife.75237

22. Boeva V, Popova T, Bleakley K, Chiche P, Cappo J, Schleiermacher G, et al. Control-FREEC: a tool for assessing copy number and allelic content using next-generation sequencing data. Bioinforma Oxf Engl. 2012 Feb 1;28(3):423–5. doi:10.1093/bioinformatics/btr670 PubMed PMID: 22155870; PubMed Central PMCID: PMC3268243.

23. Sanchez L, Messenger LA, Bhattacharyya T, Gilman RH, Mayta H, Colanzi R, et al. Congenital Chagas disease in Santa Cruz Department, Bolivia, is dominated by Trypanosoma cruzi lineage V. Trans R Soc Trop Med Hyg. 2021 Jun 16;116(1):80–4. doi:10.1093/trstmh/trab089 PubMed PMID: 34134129; PubMed Central PMCID: PMC8776560.

24. Bowman NM, Balasubramanian S, Gilman RH, Parobek C, Calderon M, Waltmann A, et al. Deep Sequencing to Detect Diversity of Trypanosoma cruzi Infection in Patients Coinfected With Human Immunodeficiency Virus and Chagas Disease. J Infect Dis. 2021 Jul 8;jiab350. doi:10.1093/infdis/jiab350

25. Hakim JMC, Waltmann A, Tinajeros F, Kharabora O, Málaga Machaca E, Calderon M, et al. Amplicon sequencing reveals complex infection in infants congenitally infected with Trypanosoma cruzi and informs the dynamics of parasite transmission. J Infect Dis. 2023 Apr 29;jiad125. doi:10.1093/infdis/jiad125

26. Talavera-López C, Messenger LA, Lewis MD, Yeo M, Reis-Cunha JL, Matos GM, et al. Repeat-Driven Generation of Antigenic Diversity in a Major Human Pathogen, Trypanosoma cruzi. Front Cell Infect Microbiol [Internet]. 2021 [cited 2022 Apr 18];11. Available from: https://www.frontiersin.org/article/10.3389/fcimb.2021.614665

27. Cosentino RO, Agüero F. A Simple Strain Typing Assay for Trypanosoma cruzi: Discrimination of Major Evolutionary Lineages from a Single Amplification Product. PLoS Negl Trop Dis. 2012 Jul 31;6(7):e1777. doi:10.1371/journal.pntd.0001777

28. da Silva MS. DNA Double-Strand Breaks: A Double-Edged Sword for Trypanosomatids. Front Cell Dev Biol. 2021 Apr 15;9:669041. doi:10.3389/fcell.2021.669041 PubMed PMID: 33937271; PubMed Central PMCID: PMC8085331.

29. Richardson C, Moynahan ME, Jasin M. Double-strand break repair by interchromosomal recombination: suppression of chromosomal translocations. Genes Dev. 1998 Dec 15;12(24):3831–42. doi:10.1101/gad.12.24.3831 PubMed PMID: 9869637; PubMed Central PMCID: PMC317271.

30. Macías F, Afonso-Lehmann R, López MC, Gómez I, Thomas MC. Biology of Trypanosoma cruzi Retrotransposons: From an Enzymatic to a Structural Point of View. Curr

Genomics. 2018 Feb;19(2):110–8. doi:10.2174/1389202918666170815150738 PubMed PMID: 29491739; PubMed Central PMCID: PMC5814959.

31. Ribeiro YC, Robe LJ, Veluza DS, dos Santos CMB, Lopes ALK, Krieger MA, et al. Study of VIPER and TATE in kinetoplastids and the evolution of tyrosine recombinase retrotransposons. Mob DNA. 2019 Aug 5;10(1):34. doi:10.1186/s13100-019-0175-2

32. Vázquez M, Ben-Dov C, Lorenzi H, Moore T, Schijman A, Levin MJ. The short interspersed repetitive element of Trypanosoma cruzi, SIRE, is part of VIPER, an unusual retroelement related to long terminal repeat retrotransposons. Proc Natl Acad Sci. 2000 Feb 29;97(5):2128–33. doi:10.1073/pnas.050578397
