# Supplementary material for: Genomic diversity of Trypanosoma cruzi clinical isolates: a retrospective analysis
Source: Lancet Microbe. Author manuscript; Available in PMC 2026 Sep 26. (PMC13615562; doi:10.1016/j.lanmic.2026.101446)
Supplement: Supplementary file 2 [file NIHMS2208885-supplement-supplementary_appendix_2.pdf]

THE LANCET
Microbe

Supplementary appendix 2

This appendix formed part of the original submission and has been peer reviewed.
We post it as supplied by the authors.

Supplement to: Hakim JM C, Guarnizo SAG, Duran A, et al. Genomic diversity of *Trypanosoma cruzi* clinical isolates: a retrospective analysis. *Lancet Microbe* 2026. https://doi.org/10.1016/j.lanmic.2026.101446

Luciana Lidia Basma Melgar, basmaluciana@gmail.com, Bolivia , Universidad Católica Boliviana "San Pablo"
Shirley Equilia, equilia.skeb@gmail.com, Bolivia Asociacion Benefica Prisma (PRISMA). Lima, Peru
Beth Jessy Condori, beth.condori.a@upch.pe, Peru, Infectious Diseases Research Laboratory, Department of Cellular and Molecular Sciences, Universidad Peruana Cayetano Heredia, Lima, Peru.
Edith Malaga, edith.malaga@gmail.com, Peru, Infectious Diseases Research Laboratory, Department of Cellular and Molecular Sciences, Universidad Peruana Cayetano Heredia, Lima, Peru.
Venilda Doris Quiñones Araujo , Nelsivenilda@gmail.com , Peru ,Infectious Diseases Research Laboratory, Department of Cellular and Molecular Sciences, Universidad Peruana Cayetano Heredia, Lima, Peru.
Emily Arteaga, emilyarteagacarrasco@gmail.com ,Bolivia Asociacion Benefica Prisma (PRISMA). Lima, Peru
Jean Karla Velarde Leigue ,velardeleiguejeankarla@gmail.com , Bolivia, Etta Projects
Martin Obregon, pepe.obregon@unmsm.edu.pe, , Infectious Diseases Research Laboratory, Department of Cellular and Molecular Sciences, Universidad Peruana Cayetano Heredia, Lima, Peru.
Freddy Tinajeros, ftinajeros@gmail.com, Bolivia Asociacion Benefica Prisma (PRISMA). Lima, Peru
Rony Pedro Colanzi Zeballos, ronypcolanzi@gmail.com , Bolivia, Hospital universitario Japonés
Clariza Roxana Chavez Velasco , clariza.ro@gmail.com, Bolivia, Etta Projects
Pamela Ontiveros Alvarado, pame181_oa@hotmail.com, Bolivia, Hospital universitario Japonés
Margot Rosse Mary Ramirez Jaldin , margotcita_rj@hotmail.com, Bolivi, Etta Projects
Monica Pajuelo Travezaño, monica.pajuelo.t@upch.pe ,Peru, Infectious Diseases Research Laboratory, Department of Cellular and Molecular Sciences, Universidad Peruana Cayetano Heredia, Lima, Peru.
Manuela Verastegui , manuela.verastegui@upch.pe , Peru, Infectious Diseases Research Laboratory, Department of Cellular and Molecular Sciences, Universidad Peruana Cayetano Heredia, Lima, Peru.
Liseth Roque Velasquez, lrv.liseth@gmail.com, Bolivia, Etta Projects
Edith Mirea Hinojosa Pérez , edith.mirea@gmail.com, Bolivia, Etta Projects
Paloma Montserrat Samamé Salazar, paloma180195@gmail.com,Bolivia , Asociación Benéfica Prisma (PRISMA), Lima Perú
Josselyn Karina Vaca , Josselyn.vaca@upch.pe, Peru, Infectious Diseases Research Laboratory, Department of Cellular and Molecular Sciences, Universidad Peruana Cayetano Heredia, Lima, Peru.

Maria Alejandra Perez Pachas, maria.perez.pachas@upch.pe, Peru, Infectious Diseases Research Laboratory, Department of Cellular and Molecular Sciences, Universidad Peruana Cayetano Heredia, Lima, Peru.
Cesar Miguel Gavidia, cgavidiac@unmsm.edu.pe, Peru, Facultad de Medicina Veterinaria, Universidad Nacional Mayor de San Marcos
Neiza Vargas Claure, neizavc@gmail.com, Asociacion Benefica Prisma (PRISMA). Lima, Peru

Franco Ignacio Soto Mercado, isotom283@gmail.com, Bolivia , Universidad Católica Boliviana "San Pablo"
Dayana Nicole Serrano Olmos, nicoleseranoolmos@gmail.com, Bolivia , Universidad Católica Boliviana "San Pablo"
Juana Maria Najarro Pucuhuayla, juana.najarro.p@upch.pe, Peru, Universidad Peruana Cayetano Heredia
Fátima Daniela Tineo Pérez, fatima.tineo@unmsm.edu.pe, Peru, Facultad de Medicina Veterinaria, Universidad Nacional Mayor de San Marcos
Yessabel Antuanet Morante Vera, yessabel.morante@unmsm.edu.pe , Peru , Universidad Peruana Cayetano Heredia
Roberto César Dávila, rdavilaf@unmsm.edu.pe,Perú, Facultad de Medicina Veterinaria, Universidad Nacional Mayor de San Marcos
Mauricio Steven Guzmán Tapia, mauriciosteven.11.7@gmail.com, Bolivia , Asociacion Benefica Prisma (PRISMA). Lima, Peru
Richard David Aguirre Barrientos, Richard.aguirre@upch.pe ,Peru, Infectious Diseases Research Laboratory, Department of Cellular and Molecular Sciences, Universidad Peruana Cayetano Heredia, Lima, Peru.
Oscar Pedro Nizama Salazar, nizamaoscar@gmail.com, Peru , Universidad Peruana Cayetano Heredia
Omar Nuñez Casio, omar.nunez@unmsm.edu.pe, Peru, "Infectious Diseases Research Laboratory, Department of Cellular and Molecular Sciences, Universidad Peruana Cayetano Heredia, Lima, Peru. Facultad de Medicina Veterinaria, Universidad Nacional Mayor de San Marcos"
